# Anti-HIV-1 activity of salivary MUC5B and MUC7 mucins from HIV patients with different CD4 counts

**DOI:** 10.1186/1743-422X-7-269

**Published:** 2010-10-14

**Authors:** Habtom H Habte, Corena de Beer, Zoë E Lotz, Paul Roux, Anwar S Mall

**Affiliations:** 1 Department of Surgery, Division of General Surgery, University of Cape Town, Observatory, Cape 7925, South Africa; 2 Discipline of Medical Virology, University of Stellenbosch and National Health Laboratory Service, Tygerberg, South Africa; 3 Department of Paediatric Medicine, University of Cape Town, Observatory, Cape 7925, South Africa

## Abstract

**Background:**

We have previously shown that MUC5B and MUC7 mucins from saliva of HIV negative individuals inhibit HIV-1 activity by 100% in an *in vitro *assay. The purpose of this subsequent study was to investigate whether MUC5B and MUC7 from saliva of HIV patients or with full blown AIDS had a similar inhibitory activity against the virus.

**Methods:**

Salivary MUC5B and MUC7 from HIV patients with different CD4 counts (< 200, 200-400 and > 400) were incubated with HIV-1 prior to infection of the human T lymphoblastoid cell line (CEM SS cells). Cells were then cultured and viral replication was measured by a qualitative p24 antigen assay. The size, charge and immunoreactivity of mucins from HIV negative and positive individuals was also analysed by SDS-PAGE, Western blot and ELISA respectively.

**Results:**

It was shown that irrespective of their CD4 counts both MUC5B and MUC7 from HIV patients, unlike the MUC5B and MUC7 from HIV negative individuals, did not inhibit HIV-1 activity. Size, charge and immunoreactivity differences between the mucins from HIV negative and positive individuals and among the mucins from HIV patients of different CD4 count was observed by SDS-PAGE, Western blot and ELISA.

**Conclusions:**

Purified salivary mucins from HIV positive patients do not inhibit the AIDS virus in an *in vitro *assay. Although the reason for the inability of mucins from infected individuals to inhibit the virus is not known, it is likely that there is an alteration of the glycosylation pattern, and therefore of charge of mucin, in HIV positive patients. The ability to inhibit the virus by aggregation by sugar chains is thus diminished.

## Background

Several *in vitro *studies have shown that human saliva inhibits the activity of Human Immunodeficiency Virus (HIV) [1-3] the causative agent of Acquired Immunodeficiency Syndrome (AIDS). We hypothesized and confirmed that salivary MUC5B and MUC7 [4], breast milk mucin (MUC1) [5] and cervical or pregnancy plug mucins [6] inhibited HIV-1 activity in an *in vitro *inhibition assay. We have also shown the inhibition of poxvirus activity by MUC1 [7]. With this in mind, we decided to investigate whether MUC5B and MUC7 from saliva of HIV patients or with full blown AIDS had a similar inhibitory activity, as the MUC5B and MUC7 from HIV negative individuals [4], against the virus. Although there have been documented cases of transmission of HIV through the exchange of oral fluids, this is indeed very rare and confined to a small group of those with ulcerations or injuries to the mucosal lining of the mouth (personal communication, David Coetzee, UCT).

In this assay a subtype D HIV-1 virus which was first isolated in February 1988 from an AIDS patient and characterised by the Department of Medical Virology, Tygerberg Hospital, Cape Town, South Africa, was used. Incubation of the virus with CEM-SS cells which expresses CD4, CXCR4, ICAM-3 and MHC class II molecules [8] results in the latter forming syncitia upon infection [9].

## Results

### Mucin preparation, purification, identification and analysis

As described by Habte *et al. *[4], the SDS-PAGE band appearance, Western blotting and amino acid analysis have shown the salivary mucins which eluted in the void and included volumes of the Sepharose CL-4B gel filtration column to be MUC5B and MUC7 respectively.

### Toxicity assay

Prior to the inhibition assay, the toxicity of salivary MUC5B and MUC7 mucins from HIV patients with CD4 count (< 200, 200-400 and > 400) to the CEM SS cells was determined by toxicity assay. As shown in Table 1, there was death of 5% of the cells when incubated with MUC5B from patients with CD4 count 200-400. Other than that, there was no toxic effect of any mucin to the CEM SS cells.

**Table 1:** Mucin toxicity

Sample	Con	CEM SS cells	% of dead cells	% of live cells
MUC5B CD4 < 200	0.9 mg	2.5 × 10^6^/ml	0	100

MUC5B CD4 < 200-400	0.9 mg	2.5 × 10^6^/ml	5	95

MUC5B CD4 < 400	0.9 mg	2.5 × 10^6^/ml	0	100

MUC7 CD4 < 200	0.9 mg	2.5 × 10^6^/ml	0	100

MUC7 CD4 < 200-400	0.9 mg	2.5 × 10^6^/ml	0	100

MUC7 CD4 < 400	0.9 mg	2.5 × 10^6^/ml	0	100

Toxicity of salivary MUC5B and MUC7 mucins from HIV patients with CD4 count (< 200, 200-400 and > 400) to the CEM SS cells.

### Inhibition assay

**Figure 1:**
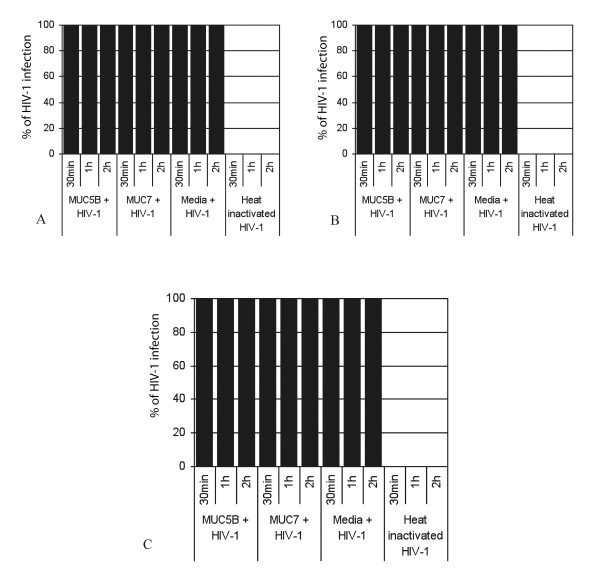
**Anti-HIV-1 activities of salivary MUC5B and MUC7 of different CD4 counts for unfiltered samples**. Salivary MUC5B and MUC7 mucins (500 μl or 0.9 mg each) from patients with CD4 count (< 200, 200-400 and > 400) were incubated with subtype D HIV-1 for 60 min and filtered through 0.45 μm pore size cellulose acetate filter. As controls HIV-1 treated with media and heat inactivated HIV-1 were used. The unfiltered samples were then incubated with CEM SS cells at a concentration of 0.5 × 10^6^cells/ml for 30 min, 1 h and 2 h. After PBS wash cells were cultured and viral replication was measured by a qualitative p24 antigen assay. Letters A, B and C indicate the anti-HIV-1 activity of salivary MUC5B and MUC7 mucins from HIV patients with CD4 counts < 200, 200-400 and > 400 respectively.

To check whether the salivary MUC5B and MUC7 mucins from HIV patients possess the same inhibitory activity as those from HIV negative individuals [4], the anti-HIV-1 activities of the salivary MUC5B and MUC7 mucins from the three groups of HIV patients (i.e. patients with CD count < 200, 200-400 and > 400) was determined in an *in vitro *inhibition assay. The result demonstrated that irrespective of their CD4 count both MUC5B and MUC7 mucins from HIV patients, unlike those HIV negative patients, failed to inhibit HIV-1 activity and a 100% viral infection of the CEM SS cells was measured by the p24 antigen assay after a 30 min incubation period (Figure 1A-C). This was unlike MUC5B and MUC7 mucins from HIV negative individuals [4]. When HIV-1 was treated with the media instead of mucins as a control, 100% HIV-1 replication or infection of the CEM SS cells was detected (Figure 1A, B and 1C). However, no HIV-1 infection was seen when heat inactivated HIV-1 was used (Figure 1A, B and 1C).

The effect of the length of the incubation period on the rate of inhibition of the HIV-1 infection of the CEM SS cells was determined by incubating the CEM SS cells with the mixtures for 1 h and 2 h. There was no difference due to the length of the incubation period (Figure 1A, B and 1C). As in the case of the MUC5B and MUC7 from HIV negative individuals [4], serial tenfold dilution, (10^-1 ^to 10^-4^) of the MUC5B and MUC7 mucins, starting at 0.9 mg mucin, from HIV patients of all the different CD4 counts (< 200, 200-400 and > 400) were done in triplicate and this made no difference to infectivity (data not shown).

**Figure 2:**
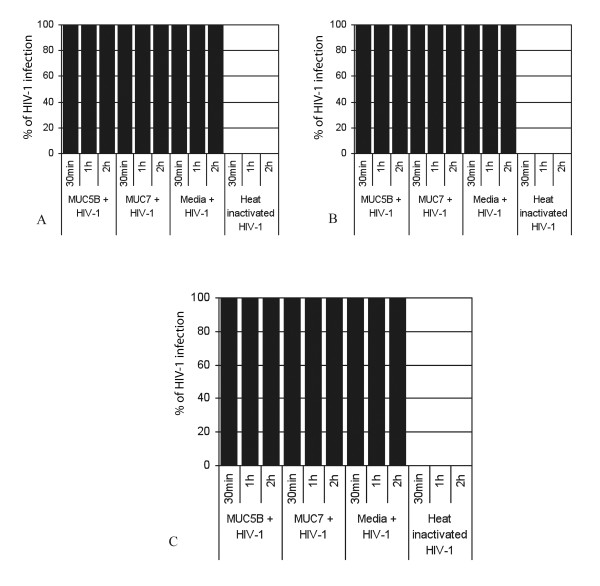
**Anti-HIV-1 activities of salivary MUC5B and MUC7 of different CD4 counts for filtrates**. The rest is as for Fig 1.

To determine if the salivary MUC5B and MUC7 mucins from HIV positive patients trap or aggregate the viruses as the MUC5B and MUC7 mucins from HIV negative individuals did [4] the mixtures were filtered through 0.45 μm pore size cellulose acetate filter at the end of the incubation period (60 min), and the filtrates were subsequently incubated with the CEM SS cells for 30 min. Unlike the filtrates from the mixtures of HIV-1 plus MUC5B and MUC7 from HIV negative individuals [4], these filtrates caused 100% viral infection of the CEM SS cells (Figure 2A, B and 2C). Even if these filtrates were incubated with the CEM SS cells for 1 h and 2 h, no change from the above results was observed (Figure 2A, B and 2C).

### Gradient gel analyses

**Figure 3:**
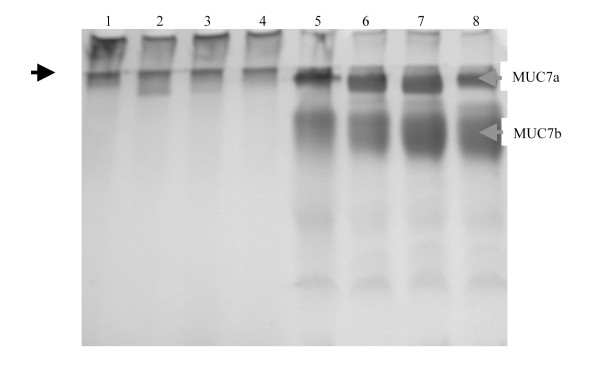
**Gradient gel analysis of salivary MUC5B and MUC7 mucins from HIV negative and positive individuals**. Freeze-dried samples (30 μg) of MUC5B from HIV negative individual (lane 1), MUC5B from HIV positive individuals with CD4 counts < 200 (lane 2), 200-400 (lane 3), > 400 (lane 4), MUC7 from HIV negative individual (lane 5), MUC7 from HIV positive individuals with CD4 counts < 200 (lane 6), 200-400 (lane 7) and > 400 (lane 8) were prepared in a gel loading buffer and separated in a 4-20% gradient gel. Following electrophoresis the gel was stained with PAS. While arrows in red indicates the two glycoforms of MUC7 on top of the running gel (MUC7a) and slightly entering the running gel (MUC7b), the arrow in black is at the start of running gel.

To assess if the HIV infection induced any structural or size difference on the salivary MUC5B and MUC7 mucins, salivary MUC5B and MUC7 mucins from HIV positive individuals with different CD4 counts (< 200, 200-400 and > 400), were dissolved in a gel loading buffer and were subjected to a 4-20% gradient gel alongside the salivary MUC5B and MUC7 mucins from HIV negative individuals as a control (Figure 3).

The PAS stained gel showed that the MUC5B from normals had slightly more material in the stacking gel and less penetration into the running gel (lane 1) than that from the HIV patients with CD4 count < 200 (lane 2), 200-400 (lane 3) and > 400 (lane 4) which showed less material in the stacking gel and high penetration into the running gel. MUC5B from patients with CD4 count < 200 (lane 2) appeared as a broader band on the top of the running gel. The MUC7a from normals had slightly more material and showed less penetration into the running gel (lane 5) than that from the HIV patients with CD4 count < 200 (lane 6), 200-400 (lane 7) and > 400 (lane 8) which showed slightly less material of MUC7a and more material of MUC7b than the normal. The MUC7a from patients with CD4 count > 400 (lane 8) showed less material than the rest.

### Immunoreactivity analyses

**Figure 4:**
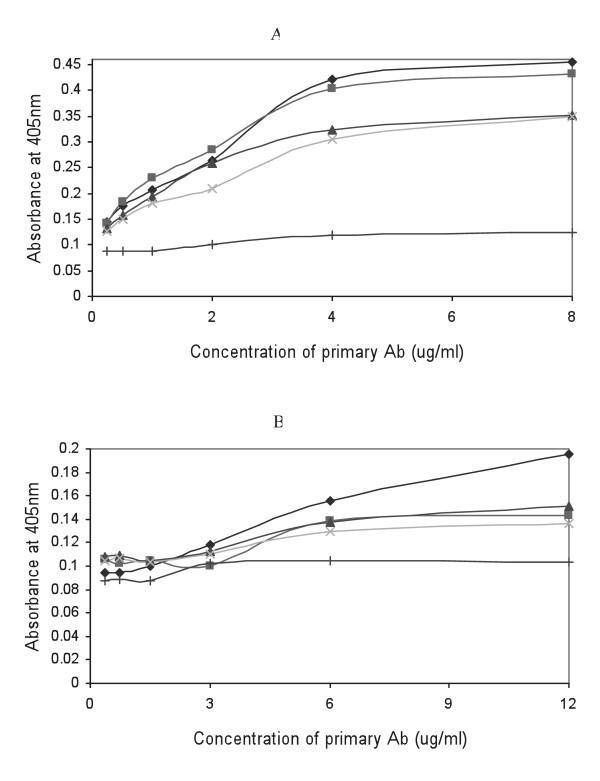
**ELISA monitoring immunoreactivity of salivary MUC5B and MUC7 mucins from HIV negative and positive individuals**. Plate (A) was coated with MUC5B from HIV negative individual (dark blue diamond), MUC5B from HIV positive individuals with CD4 counts < 200 (pink square), 200-400 (blue triangle) and > 400 (pale blue cross) and plate (B) was coated with MUC7 from HIV negative individual (dark blue diamond), MUC7 from HIV positive individuals with CD4 counts < 200 (pink square), 200-400 (blue triangle) and > 400 (pale blue cross). Plates were incubated with serial two-fold dilutions of goat anti-MUC5B (A) and goat anti-MUC7 (B) polyclonal antibodies at concentrations between 8 μg/ml and 0.25 μg/ml (goat anti-MUC5B) and 12 μg/ml and 0.375 μg/ml (goat anti-MUC7). Antibody binding was detected using rabbit anti-goat HRPO linked secondary antibody and visualized with TMB/H2O2 substrate. Absorbance values were read at 405 nm in a Titertek ELISA reader. Each point is the average absorbance of duplicate samples. As a negative control wells were coated with PBS (+).

To determine if there are any immunoreactivity differences between salivary MUC5B and MUC7 mucins from HIV negative and HIV positive individuals towards the same antibodies as the result of the HIV infection, salivary MUC5B and MUC7 mucins from HIV positive individuals with different CD4 counts (< 200, 200-400 and > 400) were coated in an ELISA plate alongside the salivary MUC5B and MUC7 from HIV negative individuals and probed with anti-MUC5B and anti-MUC7 polyclonal antibodies (Figure 4).

Although the difference in immunoreactivity between mucins from HIV negative and positive samples is very small, MUC5B (Figure 4A) and MUC7 (Figure 4B) from HIV negative individuals have shown the highest reactivity towards their respective antibodies. Interestingly, we detected immunoreactivity differences between the mucins from HIV patients of different CD4 counts as well. However, none of these differences were significant.

### Western blotting analyses

**Figure 5:**
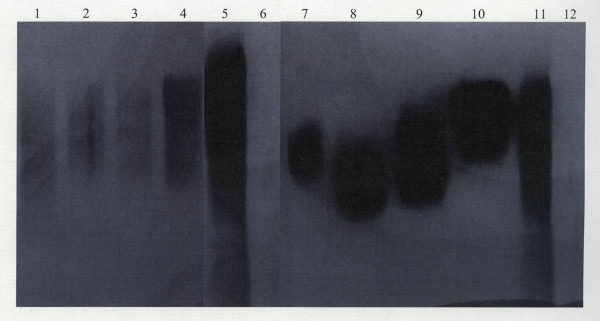
**Western blotting of salivary MUC5B and MUC7 from HIV positive individuals with different CD4 counts**. Lane 1, MUC5B from patients with CD4 > 400, lane 2, MUC5B from patients with CD4 200-400, lane 3, MUC5B from patients with CD4 < 200, lane 4, MUC5B from HIV negative individual, lane 5, crude saliva (positive control), lane 6, MUC7 (negative control), lane 7, MUC7 from patients with CD4 > 400, lane 8, MUC7 from patients with CD4 200-400, lane 9, MUC7 from patients with CD4 < 200, lane 10, MUC7 from HIV negative individuals, lane 11, crude saliva (positive control) and lane 12, MUC5B (negative control) were separated by a 1% agarose gel and transferred to nitrocellulose membrane. Following overnight blocking, the membranes were incubated for 2 h with rabbit anti-MUC5B polyclonal (lanes 1-6) and mouse anti-MUC7 monoclonal (lanes 7-12) antibodies diluted in 5% (m/v) low fat milk powder in TBST at 1 in 2000 (rabbit anti-MUC5B) and 1 in 1000 (mouse anti-MUC7). Membranes were then washed 3 × 5 min with TBST and incubated for 1 h with HRPO linked goat anti-rabbit (lanes 1-6) and goat anti-mouse (lanes 7-12) secondary antibodies diluted in 5% (m/v) low fat milk powder in TBST at dilutions of 1 in 5000 and 1 in 1500 respectively. After another TBST wash (3 × 5 min), bands were detected using an ECL detection kit.

To determine if there are any charge differences between the salivary MUC5B and MUC7 mucins from HIV negative and HIV positive individuals or among the mucins from HIV patients of different CD4 counts, MUC5B and MUC7 mucins from HIV positive individuals with different CD4 counts (< 200, 200-400 and > 400) were run in an agarose gel alongside the MUC5B and MUC7 from HIV negative individuals as a control. Mucins were then transferred to nitrocellulose membranes and probed with polyclonal rabbit anti-MUC5B and monoclonal mouse anti-MUC7 antibodies (Figure 5).

The MUC5B from HIV negative individual (lane 4) clearly had more material and a wide range of charge after equal loading, than the MUC5B from the HIV positive patients with CD4 count > 400 (lane 1), 200-400 (lane 2) and < 200 (lane 3) which showed a relatively small range of charge. The mobilities between groups hardly differed (lanes 1-5).

Differences in the charge was observed between the MUC7 from HIV negative and HIV positive individuals and within the group of HIV patients of different CD4 counts (lanes 7-10). MUC7 from HIV negative individuals (lane 10) was of lower charge than the MUC7 from HIV positive patients with CD4 count > 400 (lane 7), 200-400 (lane 8) and < 200 (lane 9). As shown in the figure, the crude saliva positive controls reacted with the anti-MUC5B and anti-MUC7 antibodies (lanes 5 and 11) as expected, whilst the negative control for MUC7 (lane 6) and for MUC5B (lane 12) did not react with the anti-MUC5B and anti-MUC7 antibodies respectively.

## Discussion

Although HIV-1 subtype C is currently the most prevalent in South Africa, the subtype D virus used in this study was found during the early stages of the HIV epidemic and is currently prevalent here, albeit less frequently. This subtype D virus is the only lab adapted strain we had available to us in the vicinity of Cape Town, in possibly the only laboratory based HIV assay in the country. The CEM-SS cells, which were used in this experiment, are known to produce distinct and repeatable syncytia formation when infected with HIV-1. These cells are capable of developing easily quantifiable syncytia formation in four to six days upon the addition of HIV-1 [9] and are reported to express CD4, CXCR4, ICAM-3 and MHC class II molecules [8], and thus could be considered a suitable model as HIV-1 host cells.

Previously we have shown that salivary MUC5B and MUC7 mucins from HIV negative individuals inhibited HIV-1 activity by 100% [4]. Here we investigated whether salivary MUC5B and MUC7 from HIV positive individuals would have a similar inhibitory effect to that from HIV negative individuals in an *in vitro *inhibitory assay. We have shown that irrespective of their CD4 count (< 200, 200-400 and > 400) both MUC5B and MUC7 mucins from HIV patients failed to inhibit HIV-1 activity. There was a 100% infection of the CEM SS cells as detected by the p24 antigen assay. Although the reason is not clear, it is possible that HIV infection induces changes of the salivary glands which results in a decline in the amount of saliva and a change in its constituents [10]. This in turn may affect the glycosylation pattern or sugar composition of the salivary mucins, and if inhibition of the virus is through aggregation by the carbohydrate side chains, and if changes in these sugar side chains occurs as a result of infection [11], it is conceivable that this is the reason for the inability of mucins from HIV positive individuals to inhibit the virus in an *invitro *assay. Furthermore HIV infection is also reported to suppress the production of saliva inhibitory factors, or elicit blocking molecules [12]. Mucins are also reported to lose their carbohydrate side chains as a result of infections which could affect their ability to adhere or recognize antigens or micro-organisms [13]. Thus there is a higher possibility that the HIV infection induced change in the glycosylation pattern of the mucins.

To check whether the HIV infection affected the ability of the mucins to trap or aggregate the virus, the filtrates of the mixtures were incubated with the CEM SS cells. The filtrates caused 100% infection of the CEM SS cells, unlike the filtrates from the mixtures of HIV-1 and normal mucins [4]. This suggests that both MUC5B and MUC7 mucins from HIV positive individuals, irrespective of their CD4 count failed to aggregate the virus. Hence there were free viruses in the filtrates that could enter the CEM SS cells and cause infection. As the carbohydrate moieties of salivary mucins serve as attachment sites for bacteria and viruses [14,15], changes in charge or glycosylation pattern as a result of HIV infection could affect the ability of the mucins to aggregate the virus.

The PAS stained 4-20% gradient gel demonstrated that there was a size difference between the mucins from HIV negative and positive individuals as well as between the mucins from HIV patients of different CD4 counts, with the mucins from HIV positive individuals showing slightly more penetration or higher electrophoretic mobility. Again changes in the glycosylation pattern could be implicated. Furthermore, as the degree of glycosylation affects the electrophoretic mobility [16,17], the appearance of the mucins from HIV negative and positive individuals on the gradient gel were different. It should be noted that any population of mucins exhibit polydispersity and even heterogeneity with respect to size, and coupled with their extensive glycosylation, they appear as smears on SDS-PAGE gels and display different electrophoretic mobilities. In summary, as the structural differences in mucins are related to physiologically different functions [18], the size or structural difference of the salivary mucins from HIV patients may affect their ability to trap or aggregate the virus. However, the differences we detected were too small to form any conclusion.

Enzyme linked immunosorbent assay (ELISA) was also performed to determine if the immunoreactivity of the salivary mucins was altered due to the HIV infection. As shown in the result section immunoreactivity differences between mucins from HIV negative and positive individuals was observed, with both MUC5B and MUC7 mucins from HIV negative individuals showing the highest reactivity. If not for the shortage of antibody, better immunoreactivity difference between MUC7 mucins from HIV negative and positive individuals could have been shown by increasing the concentration of the primary antibody. Although the reason is not clear, as variation in glycosylation can affect antibody binding [17], the HIV infection may have affected the glycosylation pattern of the mucins which could result in epitope modification.

Western blotting has shown varying mobility of MUC5B and especially MUC7 from normals and HIV patients of different CD4 counts on an agarose gel. Mucins of higher charge migrate further into the gel than those of lower charge [19]. The MUC7 from positive patients migrated further than that from normal individuals. Therefore, these diseased species of MUC7 were more highly charged than normal MUC7. If this is due to an altered glycosylation then one can expect shorter side chains and an exposure of more charged groups on the mucin, with a consequent increase in the charge per density ratio of the mucin. It would also be expected that MUC7 from patients with a CD4 count > 400 would migrate closer to where normal MUC7 is positioned. Surprisingly this was not to be the case because it seemed like the mobility of MUC7 from patients with CD4 < 200 (Figure 5, lane 9) migrated less than CD4 200-400 (lane 8) and CD4 > 400 (lane 7). Also the gels could be overloaded and thus we have not highlighted the mobility of the different mucins adequately.

The role of salivary MUC5B and MUC7 in protecting the oral cavity from bacteria, viruses, yeasts, and toxins is well documented [14,20-22]. However, Lal *et al. *[11] reported that compared to HIV negative individuals, saliva from HIV positive individuals possess considerably lower anti-candicidal activity. This was supported by the findings of Gururaja *et al. *[23] and Situ *et al. *[24] that fungal infections specifically *Candida albicans *has increasingly colonized the oral cavity of HIV positive patients. This suggested that the HIV infection may have induced functional alteration on the salivary mucins which are very potent in normal circumstances. For instance MUC7 in immunocompromised individuals is reported to lose the expression of sugar receptor (sLe^x^) hence making the individual more susceptible to oral diseases [25]. Furthermore, as covalently modified MUC7 is reported to lose its potency against *Pseudomonas aeruginosa*, *Staphylococcus aureus *and *Candida albicans *[20], the idea that HIV infection might induce structural changes to the mucins is perhaps a possibility. In summary the SDS-PAGE (gradient gel), ELISA and Western blot analysis strengthens these findings.

A limitation to this study was that of numbers in terms of patient recruitment, volume of saliva obtained from individual patients and the disruption of clinical services in a busy clinic with limited resources. The yield of MUC5B and MUC7 from a single sample was far too little and saliva samples from ten donors "for each CD4 group" had to be pooled to a final volume of approximately 10.0 ml for each group in this study. A broader study is required for statistical verifiability. The use of different HIV-1 strains in the *in vitro *assay is also necessary.

## Conclusions

In summary, irrespective of their CD4 count (< 200, 200-400 and > 400) both MUC5B and MUC7 mucins from HIV patients did not inhibit HIV-1 activity. Size, charge and immunoreactivity differences between the mucins from HIV negative and positive individuals and among the mucins from HIV patients of different CD4 count was observed by gradient gel, ELISA and Western blot.

## Methods

### Ethics

The University of Cape Town Research and Ethics Committee approved this study (ethics approval number REC REF: 283/2004).

### Materials

The CEM SS cells were from AIDS Research and Reference Reagent Programme (Germantown, USA). The p24 antigen kit was from Vironostika HIV-1 Antigen kit Biomérieux (France). Monoclonal mouse anti-MUC7 (EU-MUC7a) was kindly provided by Dallas Swallows (University College London, UK). Polyclonal rabbit anti-MUC5B (LUM5B-2) and goat anti-rabbit horse radish peroxidise (HRPO) linked secondary antibodies were kindly provided by Sara Kirkham (Manchester, UK). Polyclonal goat anti-MUC5B (sc-23024), anti-MUC7 (sc-16918) and rabbit anti-goat HRPO linked secondary antibodies were from Santa Cruz (California). Goat anti-mouse HRPO linked secondary antibody was from Novocastra (Newcastle, UK).

### Saliva collection

Saliva was collected from HIV positive female volunteers from the clinic of infectious diseases in Groote Schuur Hospital, Cape Town, South Africa. The production of saliva was stimulated by chewing on parafilm and collected into 10 ml of 6 M GuHCl containing a cocktail of protease inhibitors such as 10 mM EDTA, 5 mM NEM, 1 mM PMSF and 0.1% CHAPS, pH 6.5. Samples were collected into cooled containers on ice and stored at -20°C. Samples were grouped into three categories according to the CD4 counts of the patients, less than 200, between 200 and 400 and greater than 400. The volume of saliva produced by each individual varied. Saliva was pooled in 3 groups according to the CD4 counts, to a final volume of approximately 10.0 ml per group. The patients with CD4 count < 200 had full blown AIDS.

### Mucin preparation, purification, identification and analysis

The preparation and purification of salivary mucins was done according to the method described by Habte *et al. *[4]. Briefly, each mucin pool was separated by gel filtration on Sepharose CL-4B and subsequently purified by caesium chloride isopycnic density gradient ultra-centrifugation [26]. Mucins were further analysed by SDS-PAGE [27], identified by Western blotting [28] and amino acid analysis [29,30]. Glycoprotein was estimated by the PAS procedure of [31] and protein according to the method of Lowry *et al. *[32].

### Enzyme linked immunosorbent assay

Salivary MUC5B and MUC7 from HIV negative and HIV positive individuals with different CD4 counts (< 200, 200-400 and > 400) were coated (10 μg/ml) in PBS (150 μl per well, overnight at 4°C). Non-specific binding of the antibodies was prevented by blocking the wells with 0.5% BSA-PBS (200 μl, 1 h at 37°C) and the plates were washed three times with PBS-Tween. Serial two-fold dilutions of primary antibodies starting from 8 μg/ml (goat anti-MUC5B) and 12 μg/ml (goat anti-MUC7) were added to the plate in 0.5% BSA-PBS and incubated (100 μl, 2 h at 37°C). The plates were washed three times with PBS-Tween and rabbit anti-goat HRPO-linked secondary antibody (1 in 5000 in 0.5% BSA-PBS) was added to each well and incubated (120 μl, 1 h at 37°C). Following three washes with PBS-Tween, 150 μl of the substrate solution (TMB in 0.15 M citrate-phosphate buffer, pH 5.0) was added to each well and the colour was allowed to develop in the dark against the background of the controls (10-15 min) and the A_405 _of each well was measured in a Titertek ELISA plate reader. As a control, wells were coated with PBS.

### Toxicity assay

The toxicity of salivary MUC5B and MUC7 from HIV positive individuals with different CD4 counts (< 200, 200-400 and > 400) to the phytohaemagglutinin (PHA) stimulated CEM SS cells was determined by toxicity assay. Briefly 500 μl of the CEM SS cells in RPMI complete containing 10% Fetal Calf Serum, 1% Penicillin/Streptomycin antibiotic, 10 μmol Fungin and 50 μmol 2-mercaptoethanol (final concentration 2.5 × 10^6 ^cells/ml) was incubated with 250 μl of IL-2 and 250 μl (0.9 mg) of MUC5B and MUC7 with different CD4 counts (< 200, 200-400 and > 400) in CO_2 _incubator for 24 h. As controls CEM SS cells with IL-2 only and IL-2 without CEM SS cells (blank) were used. After spinning at 200 g for 5 min cells were resuspended in 500 μl of RPMI and live and dead cells were counted using Trypan blue exclusion criteria. The percentage of viable cells was calculated as live cells/total cells × 100.

### HIV inhibition assay

The anti-HIV-1 activities of salivary MUC5B and MUC7 from HIV positive individuals were tested in an inhibition assay according to the method of Nagashunmugam *et al. *[33]. Briefly MUC5B and MUC7 with different CD4 counts (< 200, 200-400 and > 400) were dissolved in 0.25% PBS and (500 μl or 0.9 mg each) were mixed with 4 ml of the subtype D HIV-1 supernatant fluid (SNF), at the same titer of virus used in the previous study [4] and incubated for 60 min at 37°C separately. As controls, heat inactivated HIV-1 or HIV-1 plus media (RPMI 1640 with 10% Fetal Calf Serum and IL-2) were used. At the end of the incubation period the mixtures (HIV-1 plus MUC5B from HIV patients with CD4 count < 200), (HIV-1 plus MUC5B from HIV patients with CD4 count between 200 and 400), (HIV-1 plus MUC5B from HIV patients with CD4 count > 400), (HIV-1 plus MUC7 from HIV patients with CD4 count < 200), (HIV-1 plus MUC7 from HIV patients with CD4 count between 200 and 400), (HIV-1 plus MUC7 from HIV patients with CD4 count > 400) and the control (HIV-1 plus media) were filtered through 0.45 μm pore size cellulose acetate filter (25 mm diameter) and both the unfiltered and filtered samples were incubated with the CEM SS cells at 37°C at a concentration of 0.5 × 10^6 ^cells/ml for 30 min, 1 h and 3 h. Cells were then washed three times with PBS to remove free virus and cultured. Supernatant fluid was harvested on Day 4 and viral replication was measured by a qualitative p24 antigen assay. Endpoints were calculated by the Reed-Muench formula and the 50% tissue culture infective dose (TCID_50_) was expressed as the highest dilution that produced a positive qualitative p24 antigen result. All samples were done in triplicate and the anti-HIV-1 activity of each sample was tested in a serial tenfold dilution (10^-1 ^to 10^-4^).

## Competing interests

The authors declare that they have no competing interests.

## Authors' contributions

HHH carried out the biochemical experiments and drafted the manuscript. CdB established and oversaw the *in vitro *assay. ZEL helped with the biochemistry experiments. PR gave clinical advice and oversaw sample collection. ASM originated the idea, supervised the project and reviewed the drafts. All authors read and approved the final manuscript
